# Nigeria: “Ground Zero" for the High Prevalence Neglected Tropical Diseases

**DOI:** 10.1371/journal.pntd.0001600

**Published:** 2012-07-31

**Authors:** Peter J. Hotez, Oluwatoyin A. Asojo, Adekunle M. Adesina

**Affiliations:** 1 Sabin Vaccine Institute and Texas Children's Hospital Center for Vaccine Development, Departments of Pediatrics (Section of Pediatric Tropical Medicine) and Molecular Virology & Microbiology, Baylor College of Medicine, Houston, Texas, United States of America; 2 National School of Tropical Medicine, Baylor College of Medicine, Houston, Texas, United States of America; 3 Department of Pathology and Microbiology, University of Nebraska Medical Center, Omaha, Nebraska, United States of America; 4 Department of Pathology & Immunology, National School of Tropical Medicine, Baylor College of Medicine, Houston, Texas, United States of America

*Among all of the African nations, Nigeria has the greatest number of people infected with neglected tropical diseases (NTDs). With the right political will, the country has sufficient resources to expand its current investments for the important work of Nigeria's NTD program.*

In a 2009 analysis of the NTDs in sub-Saharan Africa, one of us identified Nigeria as the country with the greatest number of cases of the so-called high prevalence NTDs, such as the intestinal helminth infections, schistosomiasis, and lymphatic filariasis (LF) [1]. Shown in Table 1 is a list of the major NTDs that can be targeted for integrated NTD control and/or elimination, which also includes onchocerciasis. The information confirms that Nigeria has the greatest number of intestinal helminth infections, i.e., ascariasis, hookworm, and trichuriasis, among all African nations, ranking fourth or fifth globally behind only the much higher populated middle-income Asian nations, such as China, India, and Indonesia [1]–[4]. Nigeria also has the greatest number of cases of schistosomiasis worldwide, with both intestinal schistosomiasis caused by *Schistosoma mansoni* and the urogenital schistosomiasis caused by *Schistosoma haematobium* endemic to that country [1], [5]. In terms of the high prevalence vector-borne NTDs, Nigeria has the greatest number of cases of LF and onchocerciasis in Africa, ranking globally third and first, respectively, and accounting for one-fourth or more of the global disease burden from these two NTDs [6]–[9]. Nigeria also has an estimated 18 million people at risk for trachoma, with nearly 1.3 million people living with trichiasis [10], and the third or fourth largest number of new cases of leprosy in Africa (behind Ethiopia and the Democratic Republic of Congo) [11], in addition to some of the greatest number of cases of the lower prevalence NTDs, including yellow fever, rabies, and Buruli ulcer in Africa [1]. The World Health Organization (WHO) reports that there may be 21 “alleged" or “suspected" remaining cases of dracunculiasis in Nigeria [12], although it is widely accepted that transmission of guinea worm has been interrupted there.

**Table 1:** Ranking of Nigeria by neglected tropical diseases cases and prevalence.

Disease	Estimated Number of cases in Nigeria	Ranking in Africa	Percentage of Global Disease Burden	Ranking Globally	Reference
Ascariasis	55 million	1	7%	5th behind India, Indonesia, China, and Bangladesh	[1]–[4]
Hookworm	38 million	1	7%	Tied for 4th with China behind India, Indonesia, and Bangladesh	[1]–[4]
Trichuriasis	34 million	1	6%	4th behind India, Indonesia, and Bangladesh	[1]–[4]
Schistosomiasis	29 million	1	14%	1	[2], [5]
Lymphatic filariasis	• 25 million• 80–121 million estimated at risk, requiring mass drug administration	1	21%	3rd	[6]–[8], [27]
Onchocerciasis	30 million at risk, requiring mass drug administration	1	36%	1	[6], [9]
Trachoma	18 million at risk	Not determined	Not determined	Not determined	[10]
Leprosy	4,531 registered prevalence	4	2%	7th	[11]

The high prevalence NTDs are responsible for an enormous disease burden in Africa, equivalent to almost one-half the disease burden from malaria when measured in disability-adjusted life years [1]. There is an equally important adverse economic impact because of the effects of these NTDs on maternal-child health and worker productivity in Africa [1]. However, the seven most common NTDs can often be controlled or in some cases even eliminated through low cost “rapid-impact" packages of drugs, which are either donated by multinational pharmaceutical companies or through the purchase of low-cost generic drugs. At a cost of less than US$1 per person annually, the prevalence of the intestinal helminth infections and schistosomiasis could be reduced in some areas, while LF, onchocerciasis, and trachoma might even be eliminated over a period of several years. Therefore, based on Nigeria's current population estimate of approximately 150 million people [13], we estimate that such goals could be achieved in Nigeria for significantly less than US$100 million annually. Because the seven high prevalence NTDs have been shown to actually cause poverty, the economic rate of return for integrated NTD control and elimination would be substantial.

The enormous disease and economic burden resulting from the seven high prevalence NTDs persist in Nigeria despite the country's economic capacity to absorb some or all of the costs required for disease control and elimination. Nigeria is the most populated nation in Africa, accounting for approximately 20% of Africa's population (Figure 1, Table 2) [6], [13], [14]. It is also the 8th most populated nation worldwide, roughly equivalent to the population of Bangladesh and Brazil [13], [14], but with a gross domestic product (GDP) and purchasing power parity that ranks it with several western European countries such as Belgium or Sweden [15]. Indeed, Nigeria has the third largest economy in Africa, behind South Africa and Egypt, ranking 32nd globally with a GDP of over US$300 billion [15], [16]. Additional estimates indicate that Nigeria is ranked among the top 20 countries globally for foreign direct investments [17]. Moreover, Nigeria is currently experiencing enormous economic growth, which exceeded 8% in 2010 [18], [19], and was almost 7% in 2011 [19].

**Figure 1:**
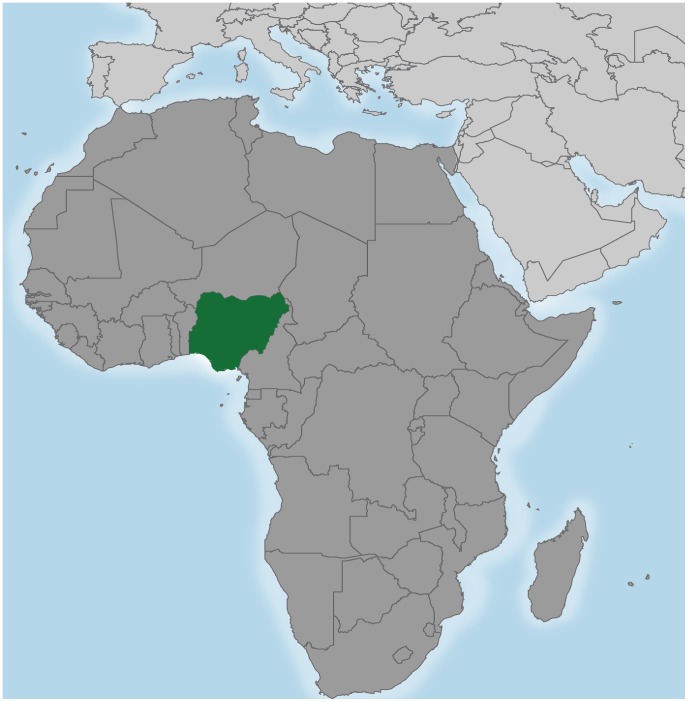
Location of Nigeria. From CIA – The World Factbook (https://www.cia.gov/library/publications/the-world-factbook/geos/ni.html), accessed 1 February, 2012.

**Table 2:** Facts and figures about Nigeria.

Category	Number	Ranking	Countries with Comparable Ranking	Reference
Population	• 152 million (2010)• 165 million (projected in 2015)	8	Bangladesh And Brazil	[13], [14]
GDP (purchasing power parity)	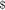 378 billion (2010)	32	Belgium andSweden	[15]
Ranking of economy in Africa		3	Behind South Africa and Egypt	[15], [16]
HDI (Human Development Index)	0.470 (2005)	158	Eritrea andTanzania	[22]

Increasingly, much of Nigeria's economic growth depends on oil and other fossil fuels. Today, Nigeria ranks 15th globally in world oil production and 5th in oil exports, in addition to 11th in natural gas exports [20]. The excessive dependence on fossil fuels has created a structural imbalance and lack of diversification leading to high youth unemployment and widespread insecurity [18]. The term “resource curse" has been applied to countries such as Nigeria that have enormous natural resources but underperform with respect to their human development index (HDI) and other metrics linked to a robust economy [21]. Indeed, despite its wealth, Nigeria ranks only 158th in terms of its HDI [22], and so far the country has been unable to meet its Millennium Development Goals (MDGs), including MDG 6 for combating HIV/AIDS, malaria, and other diseases [13]. Additionally, between the years 1990 and 2006, Nigeria regressed in several developmental indices, notably the percentage of its population that had access to safe water coverage and basic sanitation. As of 2006, a majority of Nigerians lack access to safe water coverage (53%) and sanitation (70%) [23]. This situation is not unique to Nigeria, but unfortunately is common among many African nations.

The good news is that Nigeria has made some important strides in NTD control and elimination [6], [24]. Some of these successes were accomplished in collaboration with the WHO, UNICEF-Nigeria, and the Atlanta-based Carter Center and its Nigerian offices in Jos, as well as other non-governmental developmental organizations (NGDOs) [6], [8], [24], [25], [26]. The clearest public health victory has been with respect to guinea worm (dracunculiasis) eradication. In 1986, Nigeria accounted for approximately 75% of the world's 3.3 million cases of dracunculiasis. Through investments by the Nigerian government that exceeded US$2 million, in addition to other public and private support, transmission of guinea worm has been halted since 2009 [6]. In addition, with support from the International Trachoma Initiative (ITI), the Nigeria national program has received more than 4.7 million Zithromax treatments since 2010 [10]. In 2011, Nigeria was scheduled to treat 3.1 million people [10], or possibly as many as 5 million [27]. Moreover, in collaboration with the African Programme for Onchocerciasis Control (APOC), the Nigerian Federal Ministry of Health has ensured that more than 96% of 35,000 Nigerian communities at risk for river blindness have received or still receive annual community-directed treatments with ivermectin (CDTI) [24] (Figure 2). These activities have occurred through support of the APOC Trust Fund, NGDOs, and the Nigerian government [24]. Outcomes of recent epidemiological assessments conducted with support from APOC indicate that onchocerciasis transmission has been halted in foci in Ebonyi, Kaduna, and Zamfara states [27]. For onchocerciasis and the other high prevalence NTDs, including LF, schistosomiasis, and trachoma, some of the most notable gains have occurred in the states of Plateau and Nasarawa, where the Carter Center maintains active programs of mass drug administration [6], [25], [26]. As a result, there has been a 95% reduction in onchocercal nodules in these two states, and an 83% reduction in the prevalence of LF [6], [25], [26]. Moreover, praziquantel mass drug administration for schistosomiasis is being integrated with LF and onchocerciasis control and elimination efforts, with targeted praziquantel treatment for schistosomiasis now ongoing in six states [6], [8], [27] (Supporting Document S1).

**Figure 2:**
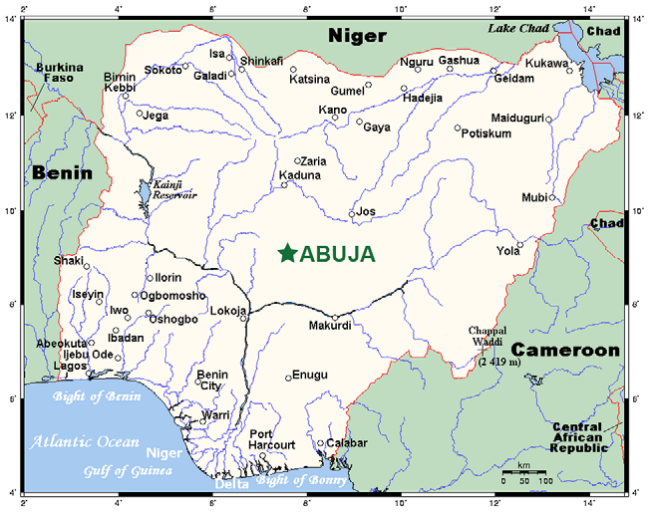
Map of Nigeria. From CIA – The World Factbook (https://www.cia.gov/library/publications/the-world-factbook/geos/ni.html), accessed 1 February, 2012.

Despite these victories and accomplishments, much of the Nigerian population still lacks access to essential medicines for the high prevalence NTDs. Beginning in 2009, a national program for NTD control and elimination was initiated to promote integrated control nationally. The NTD program of the Nigerian Federal Ministry of Health currently addresses the following diseases: LF, onchocerciasis, schistosomiasis, soil-transmitted helminth infections, trachoma, leprosy, Buruli ulcer, human African trypanosomiasis, and guinea worm disease. The strategy of the NTD program is to progressively reduce morbidity, disability, and mortality due to NTDs using integrated and cost-effective approaches with the goal to eliminate NTDs in Nigeria by the year 2020 [27].

Nigeria has sufficient wealth and resources in order to build on its past successes and embark on an expanded and aggressive program of national disease control and elimination for its highest prevalence NTDs. Costing less than 0.1% of its GDP annually, the program would be comprised of integrated mass drug administration efforts to target the intestinal helminth infections, schistosomiasis, LF, and onchocerciasis, possibly together with administration of long-lasting insecticide-treated nets to also target malaria and LF, as well as the SAFE (surgery, azithromycin antibiotics, facial cleanliness, and environmental control) strategy for trachoma elimination. Through its long-standing collaborations with international agencies such as APOC, WHO, and UNICEF, as well as CBM, ITI, the Carter Center, Helen Keller International, Sightsavers International, Mission to Save the Helpless (MITOSATH), and other NGDOs, the Nigerian Federal Ministry of Health has acquired deep and lasting technical expertise in order to ensure such expanded activities are conducted at maximal efficiencies, minimal costs, and with adequate monitoring and evaluation. A key component would include accelerated mapping of NTDs in Nigeria [28], including an ongoing partnership with the Swiss Tropical and Public Health Institute [29]. A national program of NTD control and elimination, coupled with increased access to clean water and sanitation, would simultaneously serve to strengthen health systems in many of the more fragile Nigerian states, and would represent a highly effective pro-poor strategy for Nigerian President Goodluck Jonathan in his first elected term. Doing so would be an appropriate activity for a nation currently undergoing its longest period of civilian rule since independence and one recently awarded a seat on the United Nations Security Council [30].

There is also an urgent need for new and improved control tools for Nigeria's NTDs, including the development of a new macrofilaricide for onchocerciasis and other medicines, simpler and less expensive diagnostic reagents, and NTD vaccines [6]. In parallel with expanded disease control and elimination efforts, Nigeria's best universities and research institutes must expand their research and training capacity for NTDs. Several Nigerian capacity-building and partnering programs with institutions in the United States and United Kingdom are already in place, including one between the Nigeria's National Academy of Science together with the US National Academies through the African Science Academy Development Initiative (ASADI) [31], and between the University of Ibadan (in partnership with several other Nigerian universities) and two US universities (Northwestern University and Harvard School of Public Health) through the Medical Education Partnership Initiative in Nigeria (MEPIN) supported by the US National Institutes of Health [32], but these too could be expanded.

A Nigeria free from its high prevalence NTDs can be expected to accelerate that nation's economic development through improvements in child growth, intellect and cognition, pregnancy outcome, and worker productivity. Through expansions in integrated NTD control and disease elimination, Nigeria would become an important role model for all of Africa.

## Supporting Information

Supporting Document S1 “Brief on NTD," unpublished document by Nigerian Ministry of Health, received January 24, 2012.
(DOC)

## References

[1] Hotez PJ, Kamath A (2009) Neglected tropical diseases in Sub-Saharan Africa: review of their prevalence, distribution, and disease burden. PLoS Negl Trop Dis 3: e412 doi:10.1371/journal.pntd.0000412. 19707588 10.1371/journal.pntd.0000412 PMC2727001

[2] DeSilva NR, Brooker S, Hotez PJ, Montresor A, Engels D, et al (2003) Soil-transmitted helminth infections: updating the global picture. Trends Parasitol 19: 547–551. 14642761 10.1016/j.pt.2003.10.002

[3] Hotez PJ, Ehrenberg JP (2010) Escalating the global fight against neglected tropical diseases through interventions in the Asia Pacific region. Adv Parasitol 72: 31–53. 20624527 10.1016/S0065-308X(10)72002-9

[4] Lobo DA, Velayudhan R, Chatterjee P, Kohli H, Hotez PJ (2011) The neglected tropical diseases of India and South Asia: review of their prevalence, distribution, and control or elimination. PLoS Negl Trop Dis 5: e1222 doi:10.1371/journal.pntd.0001222. 22039553 10.1371/journal.pntd.0001222 PMC3201909

[5] Steinmann P, Keiser J, Bos R, Tanner M, Utzinger J (2006) Schistosomiasis and water resources development: systematic review, meta-analysis, and estimates of people at risk. Lancet Infect Dis 6: 411–425. 16790382 10.1016/S1473-3099(06)70521-7

[6] Njepuome NA, Hopkins DR, Richards FO Jr, Anagbogu IN, Pearce PO, et al (2009) Nigeria's war on terror: fighting dracunculiasis, onchocerciasis, lymphatic filariasis, and schistosomiasis at the grassroots. Am J Trop Med Hyg 80: 691–698. 19407107

[7] Lindsay SW, Thomas CJ (2000) Mapping and estimating the population at risk from lymphatic filariasis in Africa. Trans R Soc Trop Med Hyg 94: 37–45. 10748895 10.1016/s0035-9203(00)90431-0

[8] The Carter Center (n.d.) Fighting disease: Nigeria. Available: http://www.cartercenter.org/countries/nigeria-health.html. Accessed 26 November 2011.

[9] Sightsavers (n.d.) Combating NTDs in Nigeria. Available: http://www.sightsaversusa.org/our_work/where_we_work/west_africa/nigeria/12814.html. Accessed 26 November 2011.

[10] International Trachoma Initiative (n.d.) Nigeria. Available: http://www.trachoma.org/nigeria. Accessed 21 January 2012.

[11] World Health Organization (2011) Leprosy update, 2011. Weekly Epidemiol Rec 86: 389–400.

[12] World Health Organization (2011) Monthly report on dracunculiasis cases, January–September 2011. Weekly Epidemiol Rec 86: 541–556.

[13] US Department of State (2011) Background note: Nigeria. Available: http://www.state.gov/r/pa/ei/bgn/2836.htm. Accessed 26 November 2011.

[14] Coutsoukis P (n.d.) Total population by country, 1950, 2000, 2015, 2025, 2050 (medium-fertility variant). http://www.photius.com/rankings/world2050_rank.html. Accessed 26 November 2011.

[15] Central Intelligence Agency (n.d.) Country comparison: GDP (purchasing power parity). The World Factbook. https://www.cia.gov/library/publications/the-world-factbook/rankorder/2001rank.html. Accessed 26 November 26.

[16] Chinweoke A (2011) Nigeria's economy beyond 2011 elections. Vanguard. January 8, 2011. Available: http://www.vanguardngr.com/2011/01/nigeria%E2%80%99s-economy-beyond-2011-elections/. Accessed 26 November 2011.

[17] United Nations Conference on Trade and Development (2009) World investment report 2009: transnational corporations, agricultural production and development. New York and Geneva: United Nations.

[18] African Economic Outlook (n.d.) Nigeria. Available: http://www.africaneconomicoutlook.org/en/countries/west-africa/nigeria/. Accessed 26 November 2011.

[19] Global Finance (n.d.) Nigeria country report. Available: http://www.gfmag.com/gdp-data-country-reports/207-nigeria-gdp-country-report.html. Accessed 26 November 2011.

[20] www.theodora.com (n.d.) Nigeria economy. Available: http://www.theodora.com/wfbcurrent/nigeria/nigeria_economy.html. Accessed 26 November 2011.

[21] Encyclopedia of Earth (2008) Resource curse. Available: http://www.eoearth.org/article/Resource_curse. Accessed 27 November 2011.

[22] Coutsoukis P (n.d.) HDI – Human Development Index 1975–2005 – country rankings. Available: http://www.photius.com/rankings/human_developement_index_1975-2005.html. Accessed 26 November 2011.

[23] UNICEF (2008) Nigeria. Water and sanitation summary sheet. Available: http://www.unicef.org/nigeria/ng_media_Water_sanitation_summary_sheet.pdf. Accessed 12 December 2011.

[24] WHO/APOC (n.d.) Country profiles: Nigeria. Available: http://www.who.int/apoc/countries/nga/en/index.html#. Accessed 21 January 2012.

[25] Richards FO, Eigege A, Miri ES, Kal A, Umaru J, et al (2011) Epidemiological and entomological evaluations after six years or more of mass drug administration for lymphatic filariasis elimination in Nigeria. PLoS Negl Trop Dis 5: e1346 doi:10.1371/journal.pntd.0001346. 22022627 10.1371/journal.pntd.0001346 PMC3191131

[26] Chen C, Cromwell EA, King JD, Mosher A, Harding-Esch EM, et al (2011) Incremental cost of conducting population-based prevalence surveys for a neglected tropical disease: the example of trachoma in 8 national programs. PLoS Negl Trop Dis 5: e979 doi:10.1371/journal.pntd.0000979. 21408130 10.1371/journal.pntd.0000979 PMC3050919

[27] Nigerian Federal Ministry of Health, Email communication, January 23, 2012.

[28] Ekpo UF, Mafiana CF, Adeofun CO, Solarin ART, Idowu AB (2008) Geographical information system and predictive risk maps of urinary schistosomiasis on Ogun State, Nigeria. BMC Infectious Diseases 8: 74. 18513442 10.1186/1471-2334-8-74 PMC2438363

[29] Hurlimann E, Schur N, Boutsika K, Stensgaard AS, Laserna de Himpsl M, et al (2011) Toward an open-access global database for mapping, control, and surveillance of neglected tropical diseases. PLoS Negl Trop Dis 5: e1404 doi:10.1371/journal.pntd.0001404. 22180793 10.1371/journal.pntd.0001404 PMC3236728

[30] www.theodora.com (2012) Countries of the world: Nigeria. Available: http://www.theodora.com/wfbcurrent/nigeria/nigeria_introduction.html. Accessed 26 November 2011.

[31] U.S. National Academies (n.d.) African Science Academy Development Initiative. Available: http://www.nationalacademies.org/asadi/index.html. Accessed 11 December 2011.

[32] PEPFAR (n.d.) Medical Education Partnership Initiative in Nigeria (MEPIN). Available: http://www.pepfar.gov/initiatives/mepi/network/nigeria/index.htm. Accessed 11 December 2011.

