# Supplementary material for: Nigeria: “Ground Zero" for the High Prevalence Neglected Tropical Diseases
Source: PLoS Negl Trop Dis. 2012 Jul 31;6(7):e1600. doi: 10.1371/journal.pntd.0001600 (PMC3409117; doi:10.1371/journal.pntd.0001600)
Supplement: Supporting Document S1 — “Brief on NTD," unpublished document by Nigerian Ministry of Health, received January 24, 2012. (DOC) [file pntd.0001600.s001.doc]

**Brief on NTD**

The NTD programme of the Federal Ministry of Health currently addresses the following diseases: Lymphatic Filariasis, Onchocerciasis, Schistosomiaisis, Soil Transmitted Helminths, Trachoma, Leprosy, Buruli ulcer, Human African Trypanosomiasis, and Guinea Worm Disease. However, only Lymphatic Filariasis, Onchocerciasis, Schistosomiaisis, Leprosy, and Guinea Worm Disease are effectively running. Others are still at the infancy. The strategic goal of the NTD programme is to progressively reduce morbidity, disability and mortality due to NTDs using integrated and cost-effective approaches with the view to eliminate NTDs in Nigeria by the year 2020.

The strategy for control, elimination and eradication of NTDs is premised on integrated approaches. This includes community awareness creation, Mass Drug Administration (MDA) in all endemic communities using existing Community Directed Intervention, early case detection, case management and transmission control. Integration is being pursued as this will produce such benefits as cost-effectiveness, improved coordination and programme management. Currently, there is limited integration with co-implementation of onchocerciasis control with lymphatic filariasis and schistosomiasis in a few places. The six NTD programmes that use preventive chemotherapy interventions are expected to utilize one or a combination of current delivery channels for mass drug administration. Such channels include: Community-Directed Intervention (CDI), School based treatment, and other Community-based approaches that target specific groups/populations..

***Briefs on Various NTDs***

## *Lymphatic Filariasis elimination:*.LF prevalence has been determined in 636 out of 774 LGAs of 36 States and FCT. Out of the mapped LGAs, 476 LGAs are endemic. About 15% of endemic LGAs are under Mass drug administration (MDA) with free donated Ivermectin and Albendazole tablets. Over 1 million treatments were effected in 2010

*Onchocerciasis control*: The disease is hyper and meso-endemic in 31 States and FCT. Nearly 27 million persons (75%) in 34,358 (96%) communities were treated in 2010. Many hard-to-reach communities are being covered. The CDTI has provided entry points for other community-based interventions such as Vitamin A supplementation, Guinea worm disease and Measles surveillance, elimination of Lymphatic Filariasis and Control of Schistosomiasis.

## *Schistosomiasis control:*Integrated Mapping/baseline surveys have been conducted in a total of nine States. Targeted treatment with praziquantel tablets is on-going in 6 States.

*Soil transmitted helminths control:* Integrated mapping for Soil Transmitted Helminths have been conducted in 4 States though not in all the LGAs. However, the National Programme has not commenced implementation of mass de-worming using Mebendazole tablets..

*Trachoma control:* So far 133 LGAs in 11 States are endemic for trachoma. Mapping is yet to be completed. Surgery of trichiasis cases, MDA with Azithromycin of entire at risk identified communities, improved water supply for personal hygiene, personal hygiene reinforcing face washing, and. Environmental Management are strategies to be utilized. As at 2010 mass treatment with Zithromax was yet to begin.

*Leprosy elimination:* This is endemic throughout all the States and FCT. Treatment is on-going but the national grade 2 disability rate as at the end of 2010 shows the disability rate of new cases is high and new case detection occurs relatively late.

*Human African Trypanosomiasis Elimination:* Active hospital-based surveillance is going on in a very limited number of places. Results obtained so far indicate that HAT is still endemic in the country and there is an urgent need to determine the current level of endemicity in the country.

*Buruli Ulcer control:* At the moment there is no vibrant Buruli ulcer control programme in Nigeria.

*Guinea worm Eradication*: Active surveillance activities are on in previously endemic but now freed villages, Nigeria is currently at the pre-certification stage.
